# A Solution to the Clearance Problem of Sacrificial Material in 3D Printing of Microfluidic Devices

**DOI:** 10.3390/mi14010016

**Published:** 2022-12-21

**Authors:** Terak Hornik, James Kempa, Jeffrey Catterlin, Emil Kartalov

**Affiliations:** Naval Postgraduate School, Monterey, CA 93943, USA

**Keywords:** 3D printing, additive manufacturing, microfluidic, embedded, microchannel, sacrificial material, clearance

## Abstract

3D-printing is poised to enable remarkable advances in a variety of fields, such as artificial muscles, prosthetics, biomedical diagnostics, biofuel cells, flexible electronics, and military logistics. The advantages of automated monolithic fabrication are particularly attractive for complex embedded microfluidics in a wide range of applications. However, before this promise can be fulfilled, the basic problem of removal of sacrificial material from embedded microchannels must be solved. The presented work is an experimental proof of principle of a novel technique for clearance of sacrificial material from embedded microchannels in 3D-printed microfluidics. The technique demonstrates consistent performance (~40–75% clearance) in microchannels with printed width of ~200 µm and above. The presented technique is thus an important enabling tool in achieving the promise of 3D printing in microfluidics and its wide range of applications.

## 1. Introduction

3D printing offers significant advantages to the fabrication of microfluidic devices [1,2,3,4,5] compared to traditional methods [6,7]. The 3D printing of complete internal microfluidic channels offers many benefits: rapid prototyping, 3D arrays, and scalable automated manufacturing. While extremely promising, 3D printing currently has notable issues that severely limit its use for microfluidic applications.

Stereolithography (SLA) 3D printers, using optical curing and using uncured resin as the sacrificial material, would optically overexpose by spillover intensity and blooming, because a square-profile optical beam is challenging to achieve in practice [8,9,10]. This phenomenon can be useful in bulk curing of homogeneous structures, but it is detrimental to printing fidelity at the intended boundary of the microchannel. At high resolution, the overexposed regions would overlap within the chip and the microchannel would not form properly. As a result, this printing method typically fails to form narrow embedded microfluidic channels designed as negative features, even though they are significantly larger than the resolution limit of the same printer for positive features. Furthermore, even if the overexposure problem is satisfactorily solved, the removal of uncured material in post-processing is a separate problem [11].

3D printers using UV-cured sacrificial material, e.g., proprietary composite wax in Stratasys printers, avoid the problem of optical overexposure. This makes them feasible for high-end small-feature microfluidic applications. However, the sacrificial material still needs to be removed to clear the microfluidic channels for their intended use. Standard-use clearance protocols typically involve altering the wax chemically and/or thermally, and then removing it mechanically. These techniques are designed and best suited for devices with high spatial accessibility to the wax. In contrast, microfluidic devices with embedded microchannels have only a few small ports to connect to the outside world. This makes clearance of sacrificial material particularly challenging in 3D-printed embedded microfluidics. Solving this critical problem is the subject of the presented work.

In some previous works, the problem is ignored or simply left unaddressed [12,13,14,15]. In an alternative approach, the problem is partly circumvented by piecemeal printing of components, which are then manipulated and assembled into final structures. For example, some methods utilize 3D printing of devices with grooves that become channels upon assembly to a substrate [11,16,17,18,19]. Other methods manually add sacrificial material to the initial partial print, then complete the printing [20]. Yet, other methods [2,6,7,12,19,21,22,23] 3D-print molds that are then used to cast the desired devices in a different material, e.g., polydimethylsiloxane (PDMS), by replication molding. Ultimately, all these workarounds employ multiple 2D architectures and require further assembly. Hence, they sacrifice the primary advantages of 3D printing in microfluidics: true monolithic 3D construction, embedded features, convenience, labor reduction, affordability, and scalability.

Another approach is to develop effectual techniques to clear the sacrificial material embedded within 3D-printed microfluidic devices. This approach is particularly promising, since it retains all the advantages offered by 3D printing. The drawback is that it is a major challenge to find a protocol that works sufficiently well and with sufficient generality. For example, the flushing fluid can be water [13,20], vegetable oil [23,24], ethanol [11,24,25], air [6,24,26] or more specialized chemicals [6,13,16,18]. Partial solutions have emerged, e.g., adding drainage holes to aid the removal [19] and incorporating the sacrificial material into the design of the channels [27]. However, these are very limited architecturally and too specialized to use as a general solution.

To our knowledge, the only report so far of a more general approach is described in Yin et al. [24]. It offered a combination of mechanical scraping, thermal treatment, and flushing with ethanol and vegetable oil. However, that protocol did not work at all with our Stratasys chips. That inspired and motivated us to come up with our own solution.

In the present paper, we describe our own general method of clearing the sacrificial wax from microfluidic devices, based on melting and draining the wax partially, to open a microfluidic pathway, followed by flushing with NaOH solution to complete the clearance. The protocol was implemented on multiple chips in arrays of microchannels of square cross-section of varied width. The fidelity and accuracy of the 3D printing was assessed through optical measurements of the printed channel widths. Then, the achieved clearance was assessed optically and reported as a function of printed channel width.

Our results indicate the method is successful and quite promising as a general solution to a critical problem in 3D printed microfluidics. Its successful application would enable considerable strides forward in many applications, such as biofuel cells [28], and artificial muscles [29,30], flexible electronics [31,32], and biomedical diagnostics tools [33,34].

## 2. Materials and Methods

*Chip Architecture.* The test chips were designed as a rectangular prism (90 × 69 × 4 mm) with 11 pairs of internal channels. Each channel pair was made up of one channel with access ports (for clearance measurements) and one without ports (for reference). The top surface was designed to have a raised rim, to enclose a layer of mineral oil to smooth out the surface for improved optical clarity.

The two lateral dimensions of each channel were set as equals and as multiples of 32 µm (the theoretical best resolution of the printer in the chosen mode). The planned widths were: 64, 96, 192, 288, 384, 512, 608, 704, 800, 896, and 992 µm. The inlet/outlet ports were designed to allow a 23-gauge steel tube to be held snugly without jamming or leaking.

**Figure 1:**
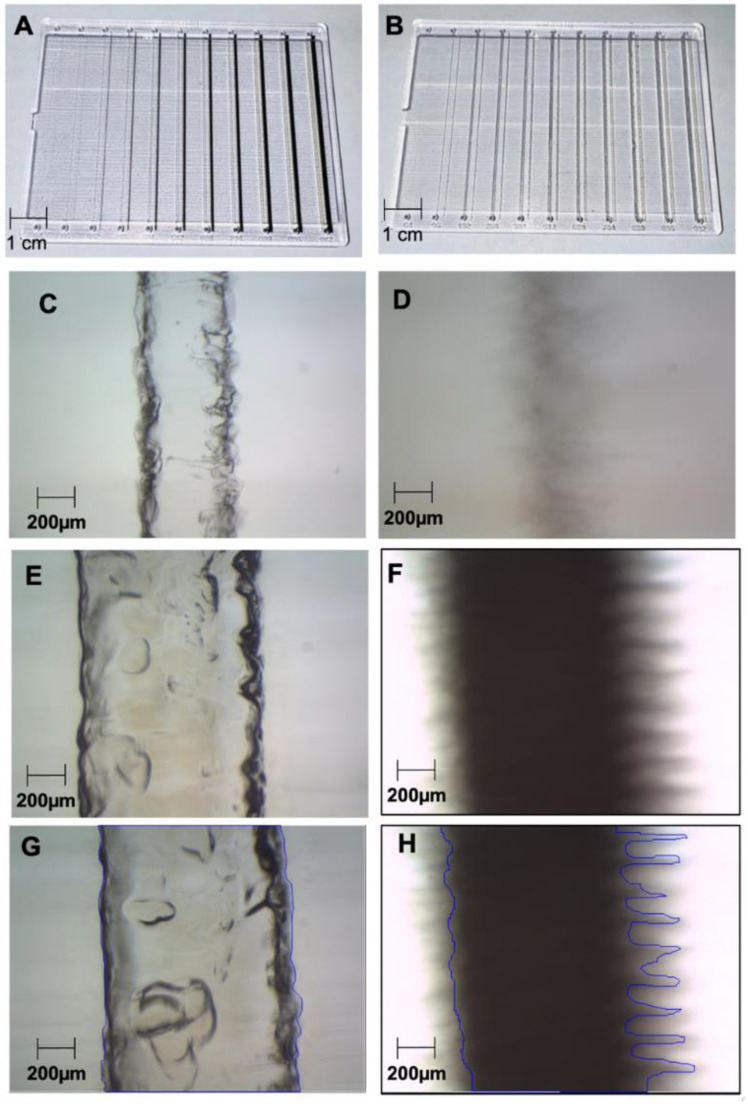
Chips and measurements. An array of flow channels was 3D-printed in wax, with matching reference channels in black resin (**A**) and wax (**B**), all encapsulated in transparent resin. All channels had a square cross-section with swept lateral dimension. The three wax/wax chips (one shown in (**B**) were put through the channel clearance procedure, while the reference chip (**A**) was not. The results were imaged in the respective pairs (**C**) (cleared flow) and (**D**) (black resin) with planned width 192 µm; (**E**) (cleared flow) and (**F**) (black resin) with planned width of 992 µm). The images were uploaded in AutoCAD and framed with polylines (**G**) (cleared flow) and (**H**) (black resin) with planned width of 992 µm). The respective framed areas were calculated through an AutoCAD function. The area ratio in each pair of images was used in the calculation of the clearance percentage.

*Fabrication.* The test chips were initially designed in SOLIDWORKS 2021 (SolidWorks Corp., Waltham, MA, USA) then converted for compatibility with our 3D printer Stratasys Objet500 Connex 1 (Stratasys Ltd., Rehovot, Israel). The digital models were then laid flat on the print bed and oriented to make regular defects inherent to the printing processes orthogonal to the channels to avoid visual distortions, then printed in batches. The Stratasys materials used were VeroClear-RGD810 for the chip itself and SUP706B for the sacrificial material. The VeroClear material was chosen due to its optical clarity, chemical resistance, and rigidity. In three chips (one shown in Figure 1B), the reference channels were printed in sacrificial material, while in a fourth one (Figure 1A), the reference channels were printed in Agilus30 Black FLX985, a black resin chosen for its optical contrast. Objet500 printed the chips monolithically, with sacrificial wax and black resin embedded in the clear resin, in a single process.

*Clearance procedure.* Sacrificial material was mechanically removed from the exterior of the chip by rubbing and scraping. The chip was sonicated for 1 h in 10% NaOH (by mass) in water. Then, the chip was rinsed in water to remove the NaOH from the outer surface. The chip was oriented to promote gravitational drainage and baked at 80 °C for a minimum of 4 h. Then, the chip was allowed to cool to room temp. A water solution of 10% NaOH was flushed through the channel by static pressure applied manually through a 1 mL syringe through a 23-gauge luer-stub adapter (Beckton-Dickinson, Franklin Lakes, NJ, USA). Then, water was similarly flushed through the channel until the exiting fluid no longer appeared milky. Then, air was similarly flushed through the channel, to remove all remaining water and help with optical detection, as air-filled portions appear darker due to refraction index mismatch.

*Optical setup and imaging*. Optical imaging was conducted on a binocular stereo 3.5–90× zoom microscope system (SM-1TZ-PL-10MA, AmScope, United Scope LLC, Irvine, CA, USA). Mineral oil (J217-500ML, VWR Life Science) was added to the top of the chips, to smooth out the surface roughness from the printing process. Multiple images from the same channel for each size for each chip were taken at the same maximal magnification using the 10 MP color camera. Figure 1 contains a collage of such images. A 10 mm calibration slide with 10 µm spacing between its bars was imaged at the same maximal magnification. This image was then used to calibrate the AmScope inbuilt software measurement tool.

**Figure 2:**
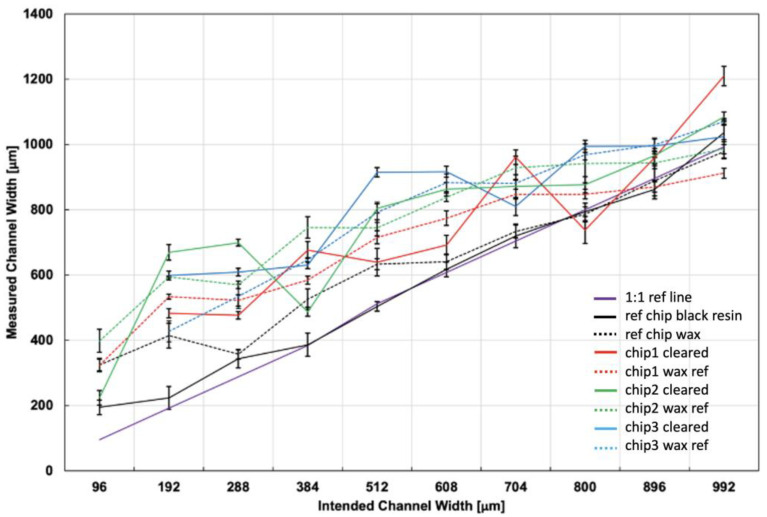
3D-print fidelity and reproducibility. Channels with square cross-section were 3D-printed in wax and in black resin, both inside bulk transparent resin. The printed physical size of each channel was measured optically at five locations. The five measurements produced an average and standard deviation, which were plotted against intended width. The results suggest high reproducibility but low fidelity for cleared flow channels and wax reference channels, particularly at the smaller dimensions, and high fidelity for black resin channels.

*Analysis of printing accuracy and reproducibility*. The calibration image was used together with the channel images to produce an absolute estimate of the channel size. For each channel, five such measurements were taken, producing a mean and a standard deviation for that channel. The results were plotted as measured width vs. intended width in Figure 2. Data from the same chip was presented in the same color. Solid color lines designated cleared flow channels. Solid black lines designated black reference channels. Trace lines designated wax reference channels.

**Figure 3:**
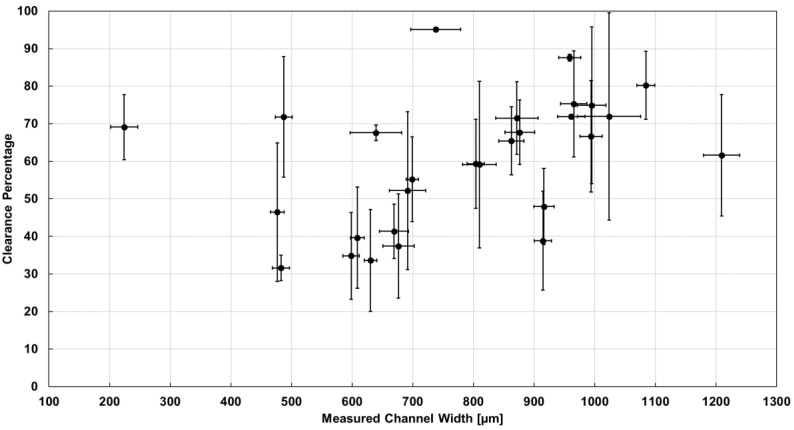
Channel clearance. The percentage clearance of each flow channel was estimated by optical measurements and plotted against its measured width. The results suggest that the procedure is successful and produces a significant clearance percentage over a wide dynamic range of square channel dimensions.

*Analysis of channel clearance.* Same-magnification images of cleared flow channels and black reference channels (Figure 1) were uploaded in AutoCAD and traced manually with polylines. The inbuilt calculator in the polyline’s Properties Palette was used to read out the area within each polyline. Each cleared area was divided by the corresponding black reference channel area, and then the result was normalized by the ratio of the average of the measured width of the black reference channel, and the average of the measured width of the cleared flow channel. The result was the percentage clearance. This parameter was obtained three times per flow channel, at different locations distributed along the channel. A corresponding average and standard deviation per flow channel were calculated and plotted in Figure 3 versus a horizontal coordinate that was the average of the measured channel widths, for the corresponding flow channel. The vertical uncertainty was produced through error propagation, while the horizontal uncertainty was the standard deviation of the multiple measurements of width for the same channel.

## 3. Results and Discussion

Microfluidics and 3D printing are both highly promising fields that stand to accomplish much when implemented together in a wide range of applications. However, this amalgamation and its benefits can only become accessible if the critical problem of clearance of the sacrificial material from embedded microchannels receives a satisfactory solution. So far, with a single arguable exception [24], such has not materialized. However, that protocol [24] did not work at all with our chips. Hence, we developed our own solution reported herein. Stratasys recommends the SUP706B sacrificial material (“wax”) to be removed by mechanical abrasion and a cleaning solution composed of NaOH and Na_2_SiO_3_. However, in microfluidics this approach at best would only clear the access ports to the microchannels, but not the microchannels themselves.

In our approach, we take this as a starting point, using sonication as our method of mechanical abrasion. The chip is then baked to partially remove the wax by melting, gravity flow, and evaporation. This clears out a pathway from the input port to the output port, which is then used to flush a NaOH solution that breaks down and removes the wax further. The 10% NaOH solutions were chosen for injection over several alternatives: Lower concentrations of NaOH were rejected as less effective, while higher concentrations were considered more dangerous to work with experimentally. Several other options, such as 2,2,4-trimethylpentane, hexane, and pentane, were attempted and proved ineffective, at least for this type of wax.

To optimize and test our clearance procedure, we designed and fabricated chips containing an array of embedded microchannels organized in pairs. Within each pair, a “flow” channel was printed with its own input and output ports, while a reference channel of the same intended size was printed without ports. In all chips, the flow channels were printed in wax. In some chips, the reference channels were printed in black resin (Figure 1A), while in others they were printed in wax (Figure 1B). This was done, because an unprocessed wax channel is very difficult to distinguish optically from the surrounding clear resin, so a flow channel could not be used reliably as its own reference to determine extent of clearance. Instead, both black resin and baked wax reference channels were used for comparison.

The channel dimensions were varied systematically in integer multiples of the theoretical best printer resolution (32 µm). Three flow channel chips (Figure 1B) underwent the clearing protocol. Then, the flow channels, the reference channels, and a calibration slide were imaged at the same magnification. Image comparisons produced an absolute measurement of printed size. For each channel, five images were taken spread out along the channel length, and their measured widths’ average and standard deviation were calculated. The results (Figure 2) were organized by intended width, in shared color for channels on the same chip. The data from cleared channels was presented in solid color line scatter plots, while the data from wax reference channels was presented in trace line scatter plots. The data from the black reference channels was presented in black solid line.

In Figure 2, the solid black scatter line and its error bars line up with the 1:1 purple line, suggesting high fidelity of printing of black resin channels, except for the smallest dimensions, where the printed channels were wider than intended. On the other hand, the wax channels were generally printed wider than intended at all dimensions (with small exceptions), so the printing accuracy is low. This is observed consistently for all wax channels, cleared and uncleared, with the largest deviation from the 1:1 happening at the smallest dimensions. The general behavior is the same regardless of which chip the data comes from, suggesting persistent effect and high reproducibility chip-to-chip.

Figure 2 shows that the untreated wax channel on the reference chip (black trace) behaved consistently with all the wax channels of the chips that underwent the procedure, both cleared and uncleared. This suggests that the clearance procedure itself was not the cause of the widening. So, the widening should stem from the printing process itself.

Figure 2 does not show results for the channels with intended width of 64 µm, because they were not observed to form. If they did form, they did not clear. Figure 1C,D show channels intended to be printed at 192 µm. The unevenness of their edges suggests that channels intended at 64 µm would likely be too irregular to form consistently. This suggests a practical limitation in horizontal/lateral resolution for the printer used.

After the clearance procedure was applied to three chips like the one in Figure 1B, images were taken at maximal magnification at three locations per channel per chip. The images were uploaded in AutoCAD. In each image, the cleared area was encompassed by a polyline and the corresponding area was calculated by an in-built function (Figure 1G). The same was done for analogous images of the black reference channel of the same intended width (Figure 1H). The results from the images of the same channel were averaged and their standard deviation was assigned as the error bars on their plotted averages.

To calculate the clearance percentage, the ratio of cleared and black areas was then adjusted by a factor equal to the average measured black reference channel width divided by the average measured cleared channel width, for the same intended width. This was done to cancel out the influence of potentially unequal fidelity in printing wax and black resin channels. Standard error propagation was applied to calculate the uncertainties on the clearance percentage. The results were plotted in Figure 3, where each datapoint represents the clearance percentage of a particular channel, plotted versus the average measured width of the same channel. Because Figure 2 suggested high reproducibility of the results across wax channels in multiple chips, all data in Figure 3 was binned together regardless of which chip it came from.

Figure 3 suggests that the clearance procedure was largely successful, as almost all channels cleared to a significant extent. The only exceptions were the ones with 64 µm intended width, which did not appear to form, and two of the three channels at 96 µm intended width, which did not clear. For most channels, the percentage clearance ranged from ~40% to ~75%. These results suggest that our clearance technique is quite effective and can be further optimized.

Once optimized, the presented technique should offer a viable solution to the critical clearance problem faced by 3D-printed embedded microfluidics, with concomitant major impact on a variety of fields that can benefit from this new capability.

## 4. Conclusions

The presented work is an experimental proof of principle of a novel technique for clearance of sacrificial material from embedded microchannels in 3D-printed microfluidics. The technique demonstrates consistent performance (~40–75% clearance) in microchannels with printed width of ~200 µm and above. The presented technique is thus an important enabling tool in achieving the promise of 3D printing in microfluidics and its wide range of applications.

## Data Availability

Not applicable.
